# Prognostic Differences and Survival Predictive Models for Mucinous Versus Usual‐Type Adenocarcinoma of the Uterine Cervix

**DOI:** 10.1002/cam4.70927

**Published:** 2025-05-02

**Authors:** Yaxin Kang, Lele Chang, Jing Liu, Haizhou Ji, Qin Xu

**Affiliations:** ^1^ Department of Radiation Oncology Clinical Oncology School of Fujian Medical University, Fujian Cancer Hospital Fuzhou Fujian Province China; ^2^ Departments of Gynecology Fujian Medical University Cancer Hospital, Fujian Cancer Hospital Fuzhou Fujian Province China

**Keywords:** adenocarcinoma, mucinous adenocarcinoma, risk prediction model, SEER database, survival analyses

## Abstract

**Background:**

There is significant histological heterogeneity between the endocervical adenocarcinoma (EA) subtypes. Usual‐type carcinoma (adenocarcinoma) and mucinous carcinoma (mucinous adenocarcinoma, MA) are the most common types of EA.

**Methods:**

Demographic and clinical variables were collected from the SEER database for selected patients between 2004 and 2021. The effect of confounding variables was reduced by propensity score matching (PSM). Survival data were analyzed using the Kaplan–Meier method and Cox regression models. A risk prediction model nomogram for MA was developed and validated.

**Results:**

The median age for MA patients was 46 years compared to 45 years for adenocarcinoma (*p* = 0.021). The 1‐, 3‐, and 5‐year overall survival (OS) rates for MA were 88.2%, 74.5%, and 68.4%, respectively, significantly lower than those for adenocarcinoma (89.0%, 79.0%, and 74.9%, *p* < 0.0001). Cancer‐specific survival (CSS) showed a similar trend (*p* < 0.0001). Seven variables, including age, primary site, T, N, combined stage, surgery, and chemotherapy, were selected to create the nomograms for predicting OS, while age, primary site, tumor size, T, N, combined stage, and surgery were selected for CSS. The validations of all predictive models were satisfactory.

**Conclusion:**

This study revealed MA's poorer prognosis compared to adenocarcinoma using the SEER database. It developed predictive models for OS and CSS of MA, offering a more accurate prognosis assessment tool for clinical practice.

## 1 Introduction

According to the International Agency for Research on Cancer (IARC), with 661,021 new cases and 348,189 deaths in 2022, cervical cancer is the fourth newly developed female malignant tumor worldwide [1]. Squamous cell carcinoma (SCC) and endocervical adenocarcinoma (EA) are the two most common subtypes of cervical cancer, contributing to about 10%–15% of cancer‐related deaths in women worldwide [1, 2]. SCC is causally related to persistent infection with high‐risk human papillomavirus (HPV). With the availability of screening and vaccines for HPV infection, the incidence of SCC has declined significantly recently [3]. Compared to SCC, 10%–15% of EA are not associated with HPV, which leads to difficulties in early prevention and screening [4, 5]. While EA is rare, the incidence has recently increased, now accounting for approximately 20%–25% of all cases [6].

EA exhibits considerable heterogeneity, characterized by the presence of a wide range of cell types and patterns [7]. Based on hematoxylin–eosin (H&E) staining results assessing morphology, mainly cytoplasmic features, the 2014 World Health Organization (WHO) Classification of cervical tumors classifies ECA into subtypes, including usual‐type carcinoma, mucinous carcinoma (mucinous adenocarcinoma, MA) (signet‐ring cell, intestinal, gastric type, not otherwise specifed [NOS]), villoglandular carcinoma, serous carcinoma, clear cell carcinoma, endometrioid carcinoma, mesonephric carcinoma, etc. [8].

Usual‐type carcinoma (adenocarcinoma) and MA, the most common EA, exhibit distinctly different histomorphological characteristics [9, 10]. However, due to the rarity, the distinctions in their clinical and prognostic features remain unclear. In this study, the cases of usual‐type endocervical adenocarcinoma and MA of the uterine cervix were extracted from the Surveillance, Epidemiology, and End Results (SEER) database to compare their prognoses.

MA is a malignant tumor originating from the columnar epithelial cells of the endocervix. Its common first symptoms are large amounts of watery or mucus‐like secretions, contact bleeding, abnormal vaginal bleeding, and abdominal pain or pelvic pain [11, 12]. Due to the lack of specificity of the clinical manifestations of MA and the hidden location of the primary site, it is not easy to be diagnosed at an early stage. Combined with the cytological features of metastasis and invasion, the clinical treatment effect is lower than that of other pathological types of cervical cancer, and the overall prognosis is relatively poor [7, 11, 13, 14]. However, there are fewer studies on the prognosis of MA. To enhance the understanding of MA, we constructed a risk prediction model. By analyzing the key prognostic indicators, it is beneficial to guide the clinical assessment of mortality risk and intervention.

## 2 Materials and Methods

### 2.1 Patient Selection

**FIGURE 1:**
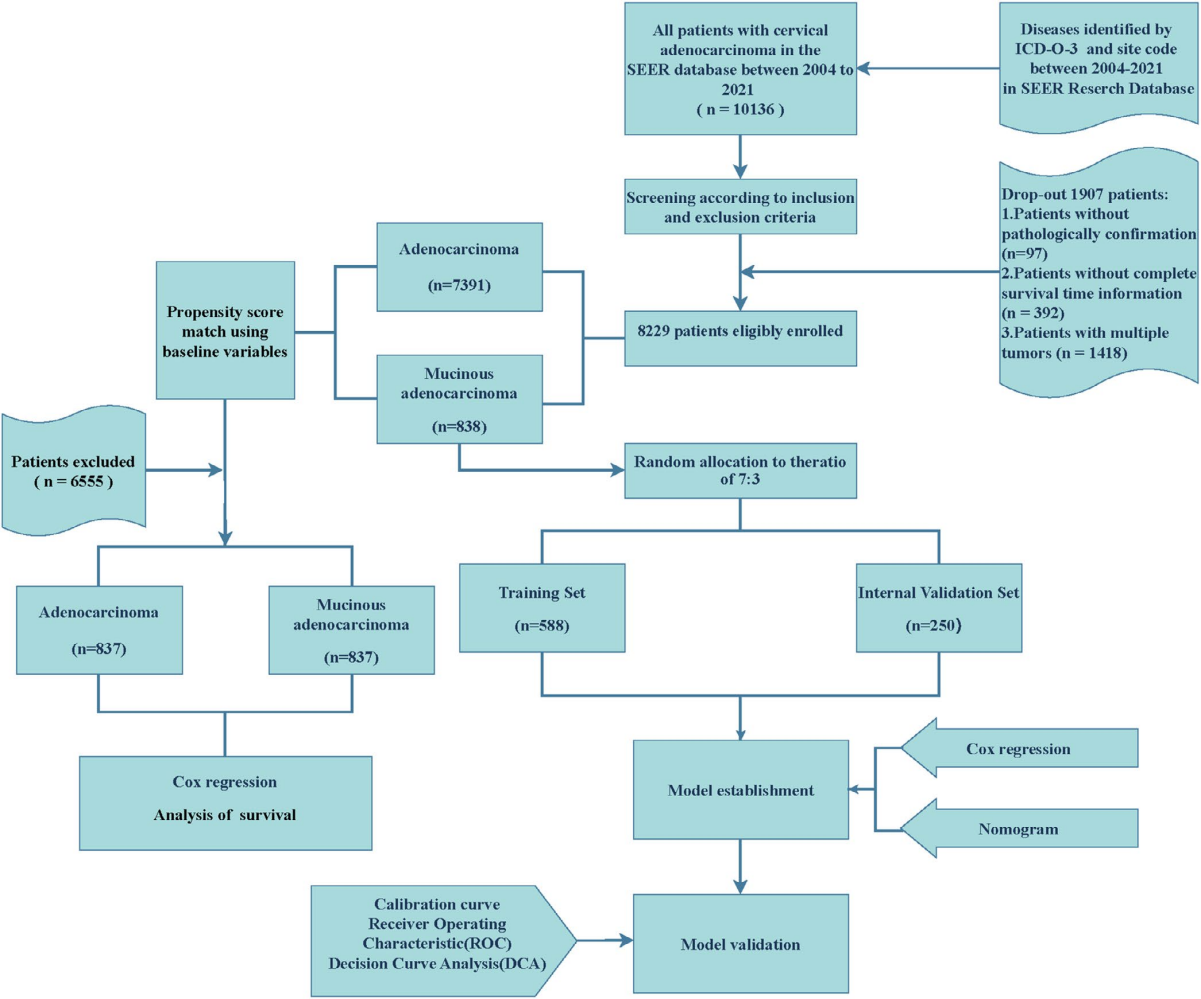
Patient inclusion and exclusion criteria and study workflow.

We systematically gathered demographic and clinical variables of patients from the SEER database (https://seer.cancer.gov/), which is publicly accessible [15]. The variables were shown in Table 1. According to the SEER Program (SEER*Stat Database: Incidence—SEER Research Data, 17 Registries, Nov 2023 Sub (2000–2021)—Linked To County Attributes—Time Dependent (1990–2022) Income/Rurality, 1969–2022 Counties, National Cancer Institute, DCCPS, Surveillance Research Program, released April 2024, based on the November 2023 submission.), patients with primary site C53.0, C53.1, C53.8, C53.9; histology codes 8140/3, 8144/3, 8480/3, 8482/3, and 8490/3 between 2004 to 2021 were included. We categorized the patients into two groups: adenocarcinoma (8140/3) and MA (, 8144/3, 8480/3, 8482/3, and 8490/3). All patients were required to have pathological confirmation and a completed survival time. Meanwhile, patients with multiple tumors were excluded. The selection process is shown in Figure 1.

**TABLE 1:** Comparison of baseline characteristics between adenocarcinoma and mucinous adenocarcinoma before and after PSM.

Characteristic	Before PSM				After PSM			
	Overall (*N* = 8229)	Adenocarcinoma (*N* = 7391)	Mucinous adenocarcinoma (*N* = 838)	*p*	Overall (*N* = 1674)	Adenocarcinoma (*N* = 837)	Mucinous adenocarcinoma (*N* = 837)	*p*
*Year of diagnosis*								
2004–2012	3530 (42.90%)	3154 (42.67%)	376 (44.87%)	0.224	753 (44.98%)	377 (45.04%)	376 (44.92%)	0.961
2013–2021	4699 (57.10%)	4237 (57.33%)	462 (55.13%)		921 (55.02%)	460 (54.96%)	461 (55.08%)	
*Race*								
White	6522 (79.26%)	5872 (79.45%)	650 (77.57%)	0.007	1304 (77.90%)	655 (78.26%)	649 (77.54%)	0.938
Black	592 (7.19%)	544 (7.36%)	48 (5.73%)		94 (5.62%)	46 (5.50%)	48 (5.73%)	
Unknown/other	1115 (13.55%)	975 (13.19%)	140 (16.71%)		276 (16.49%)	136 (16.25%)	140 (16.73%)	
*Age*	45 (38, 56)	45 (38, 56)	46 (39, 57)	0.021	46 (38, 56)	46 (38, 56)	46 (39, 57)	0.665
*Age_group*								
< 50 years	5020 (61.00%)	4534 (61.34%)	486 (58.00%)	0.060	11 (0.66%)	8 (0.96%)	3 (0.36%)	0.130
≥ 50 years	3209 (39.00%)	2857 (38.66%)	352 (42.00%)		1663 (99.34%)	829 (99.04%)	834 (99.64%)	
*Origin*								
Non‐Spanish‐Hispanic‐Latino	6218 (75.56%)	5573 (75.40%)	645 (76.97%)	0.317	1301 (77.72%)	657 (78.49%)	644 (76.94%)	0.445
Spanish‐Hispanic‐Latino	2011 (24.44%)	1818 (24.60%)	193 (23.03%)		373 (22.28%)	180 (21.51%)	193 (23.06%)	
*Marital status*								
Married	4172 (50.70%)	3770 (51.01%)	402 (47.97%)	0.194	818 (48.86%)	417 (49.82%)	401 (47.91%)	0.440
Others	3532 (42.92%)	3157 (42.71%)	375 (44.75%)		746 (44.56%)	371 (44.32%)	375 (44.80%)	
Unknown	525 (6.38%)	464 (6.28%)	61 (7.28%)		110 (6.57%)	49 (5.85%)	61 (7.29%)	
*Metropolitan or not*								
Metropolitan	7458 (90.63%)	6696 (90.60%)	762 (90.93%)	0.753	1529 (91.34%)	767 (91.64%)	762 (91.04%)	0.664
Others	771 (9.37%)	695 (9.40%)	76 (9.07%)		145 (8.66%)	70 (8.36%)	75 (8.96%)	
*Income($)*								
< 60,000	1062 (12.91%)	962 (13.02%)	100 (11.93%)	0.338	208 (12.43%)	108 (12.90%)	100 (11.95%)	0.749
60,000–70,000	1209 (14.69%)	1090 (14.75%)	119 (14.20%)		236 (14.10%)	117 (13.98%)	119 (14.22%)	
70,000–80,000	2136 (25.96%)	1932 (26.14%)	204 (24.34%)		426 (25.45%)	222 (26.52%)	204 (24.37%)	
80,000–90,000	1326 (16.11%)	1181 (15.98%)	145 (17.30%)		291 (17.38%)	147 (17.56%)	144 (17.20%)	
90,000–100,000	1115 (13.55%)	1005 (13.60%)	110 (13.13%)		213 (12.72%)	103 (12.31%)	110 (13.14%)	
100,000+	1381 (16.78%)	1221 (16.52%)	160 (19.09%)		300 (17.92%)	140 (16.73%)	160 (19.12%)	
*Primary site*								
Endocervix	4334 (52.67%)	3869 (52.35%)	465 (55.49%)	0.084	920 (54.96%)	455 (54.36%)	465 (55.56%)	0.623
Others	3895 (47.33%)	3522 (47.65%)	373 (44.51%)		754 (45.04%)	382 (45.64%)	372 (44.44%)	
*Tumor size*								
≤ 20 mm	2493 (30.30%)	2263 (30.62%)	230 (27.45%)	< 0.001	474 (28.32%)	244 (29.15%)	230 (27.48%)	0.446
20 mm–40 mm	1422 (17.28%)	1237 (16.74%)	185 (22.08%)		381 (22.76%)	196 (23.42%)	185 (22.10%)	
> 40 mm	1730 (21.02%)	1510 (20.43%)	220 (26.25%)		440 (26.28%)	221 (26.40%)	219 (26.16%)	
Unknown	2584 (31.40%)	2381 (32.21%)	203 (24.22%)		379 (22.64%)	176 (21.03%)	203 (24.25%)	
*Grade*								
Well and moderately differentiated	4254 (51.70%)	3715 (50.26%)	539 (64.32%)	< 0.001	1100 (65.71%)	562 (67.14%)	538 (64.28%)	0.199
Poorly differentiated and undifferentiated	1277 (15.52%)	1154 (15.61%)	123 (14.68%)		251 (14.99%)	128 (15.29%)	123 (14.70%)	
Unknown	2698 (32.79%)	2522 (34.12%)	176 (21.00%)		323 (19.30%)	147 (17.56%)	176 (21.03%)	
*T*								
I–II	6811 (82.77%)	6118 (82.78%)	693 (82.70%)	< 0.001	1405 (83.93%)	713 (85.19%)	692 (82.68%)	0.332
III–IV	954 (11.59%)	827 (11.19%)	127 (15.16%)		233 (13.92%)	106 (12.66%)	127 (15.17%)	
Unknown	464 (5.64%)	446 (6.03%)	18 (2.15%)		36 (2.15%)	18 (2.15%)	18 (2.15%)	
*N*								
N0	6332 (76.95%)	5714 (77.31%)	618 (73.75%)	< 0.001	1234 (73.72%)	617 (73.72%)	617 (73.72%)	0.828
N1	1376 (16.72%)	1194 (16.15%)	182 (21.72%)		359 (21.45%)	177 (21.15%)	182 (21.74%)	
Unknown	521 (6.33%)	483 (6.53%)	38 (4.53%)		81 (4.84%)	43 (5.14%)	38 (4.54%)	
*M*								
M0	7198 (87.47%)	6499 (87.93%)	699 (83.41%)	< 0.001	1395 (83.33%)	696 (83.15%)	699 (83.51%)	0.908
M1	901 (10.95%)	772 (10.45%)	129 (15.39%)		257 (15.35%)	129 (15.41%)	128 (15.29%)	
Unknown	130 (1.58%)	120 (1.62%)	10 (1.19%)		22 (1.31%)	12 (1.43%)	10 (1.19%)	
*Summary stage*								
I–II	6427 (78.10%)	5801 (78.49%)	626 (74.70%)	< 0.001	1268 (75.75%)	642 (76.70%)	626 (74.79%)	0.547
III–IV	1445 (17.56%)	1245 (16.84%)	200 (23.87%)		380 (22.70%)	181 (21.62%)	199 (23.78%)	
Unknown	357 (4.34%)	345 (4.67%)	12 (1.43%)		26 (1.55%)	14 (1.67%)	12 (1.43%)	
*Combined stage*								
Localized	4872 (59.21%)	4447 (60.17%)	425 (50.72%)	< 0.001	880 (52.57%)	455 (54.36%)	425 (50.78%)	0.476
Regional	2074 (25.20%)	1811 (24.50%)	263 (31.38%)		500 (29.87%)	237 (28.32%)	263 (31.42%)	
Distant	1002 (12.18%)	858 (11.61%)	144 (17.18%)		283 (16.91%)	140 (16.73%)	143 (17.08%)	
Unknown/others(in situ)	281 (3.41%)	275 (3.72%)	6 (0.72%)		11 (0.66%)	5 (0.60%)	6 (0.72%)	
*FIGO stage (2018)*								
Before IIB	5277 (64.13%)	4798 (64.92%)	479 (57.16%)	< 0.001	978 (58.42%)	499 (59.62%)	479 (57.23%)	0.495
IIB and after IIB	2497 (30.34%)	2162 (29.25%)	335 (39.98%)		645 (38.53%)	311 (37.16%)	334 (39.90%)	
Unknown	455 (5.53%)	431 (5.83%)	24 (2.86%)		51 (3.05%)	27 (3.23%)	24 (2.87%)	
*Surgery*								
No	2555 (31.05%)	2365 (32.00%)	190 (22.67%)	< 0.001	372 (22.22%)	182 (21.74%)	190 (22.70%)	0.638
Yes	5674 (68.95%)	5026 (68.00%)	648 (77.33%)		1302 (77.78%)	655 (78.26%)	647 (77.30%)	
*Chemotherapy*								
No/unknown	5218 (63.41%)	4780 (64.67%)	438 (52.27%)	< 0.001	888 (53.05%)	450 (53.76%)	438 (52.33%)	0.557
Yes	3011 (36.59%)	2611 (35.33%)	400 (47.73%)		786 (46.95%)	387 (46.24%)	399 (47.67%)	
*Radiation*								
No/unknown	4957 (60.24%)	4534 (61.34%)	423 (50.48%)	< 0.001	866 (51.73%)	443 (52.93%)	423 (50.54%)	0.328
Yes	3272 (39.76%)	2857 (38.66%)	415 (49.52%)		808 (48.27%)	394 (47.07%)	414 (49.46%)	

Abbreviations: FIGO, International Federation of Gynecology and Obstetrics; PSM, Propensity Score Matching.

### 2.2 Survival Analysis

The Kaplan–Meier methods were employed to visualize the changes in survival between the two groups before and after propensity score matching (PSM), and then the log‐rank tests were used to evaluate the differences in overall survival (OS) and cancer‐specific survival (CSS) between the two groups. Variables significantly associated with outcomes in univariate Cox analyses were included in a stepwise Cox regression model to identify independent predictors of adenocarcinoma and MA, separately. We also compared the prognosis between the two groups in different summary stage subgroups. OS was defined as the time from diagnosis to death from any cause, and CSS was defined as the time from diagnosis to death due to tumor.

### 2.3 Development and Validation of Risk Prediction Model for MA

Patients with MA initially included in the study from the SEER database were randomly divided into a training set and a validation set in a 7:3 ratio by “caret” package. By conducting univariate Cox analysis on the training set, we screened out variables with a *p*‐value ≤ 0.05. Subsequently, these variables were included in the multivariate Cox regression model, and variables with significant multiple factors were used to establish the model. The nomograms constructed to predict OS and CSS in MA patients were determined using stepwise backward regression with the criterion of minimizing the AIC value. We used calibration curves, time‐dependent receiver operating characteristics (time‐ROC) curves, and clinical decision analysis (DCA) curves to evaluate the model's performance. In addition, we collected patients with MA from Fujian Cancer Hospital for validation.

### 2.4 Statistical Analysis

Descriptive statistics for categorical variables are expressed as frequencies (percentage) and for continuous variables with a non‐normal distribution as medians [interquartile range (IQR)]. Chi‐squared tests were used for categorical variables and Mann–Whitney *U* Tests for continuous variables to compare the characteristics between adenocarcinoma and MA. To mitigate the effects of confounding variables, we matched patients with PSM. The PSM model was matched 1:1 based on year of diagnosis, race, age, origin, marital status, metropolitan or not, income, primary site, tumor size, grade, T, N, M, combined stage, International Federation of Gynecology and Obstetrics (FIGO) stage (2018), surgery (surgery for the primary site of cervical cancer), radiotherapy, and chemotherapy using a nearest‐neighbor matching method, with a caliper width of 0.2. A bilateral *p*‐value of < 0.05 was considered a statistically significant difference. All analyses were carried out using R (version 4.3.2) and SPSS (version 25.0).

## 3 Results

### 3.1 Clinicopathological Features of Two Groups of Patients Before PSM

In this study, we analyzed data from 8229 cervical cancer patients, of which 7391 were diagnosed with adenocarcinoma and 838 with MA. While the two groups were consistent in terms of diagnosis years (*p* = 0.224), marital status (*p* = 0.194), metropolitan or not (*p* = 0.753), and income levels (*p* = 0.338), significant differences emerged in other key variables. Racial composition showed slight variations, with white individuals constituting 79.45% of the adenocarcinoma group versus 77.57% in the MA group (*p* = 0.007). The median age was 45 for adenocarcinoma and 46 for MA (*p* = 0.021), indicating a slightly older population in the latter.

Regarding tumor biological characteristics, the MA group exhibited a higher proportion of tumors larger than 40 mm (26.25% vs. 20.43%, *p* < 0.001) and a higher percentage of well to moderately differentiated tumors (64.32% vs. 50.26%, *p* < 0.001). Moreover, more advanced TNM stages were more common in MA, including T3–T4 (15.16% vs. 11.19%, *p* < 0.001), N1 (21.72% vs. 16.15%, *p* < 0.001), M1 (15.39% vs. 10.45%, *p* < 0.001), and stages III–IV in overall staging (23.87% vs. 16.84%, *p* < 0.001).

In terms of treatment, MA patients were significantly more likely to receive surgery (77.33% vs. 68.00%, *p* < 0.001), chemotherapy (47.73% vs. 35.33%, *p* < 0.001), and radiation therapy (49.52% vs. 38.66%, *p* < 0.001). Although adenocarcinoma and MA share many demographic characteristics, they differ significantly in tumor biology, clinical progression, and treatment response. These differences underscore the importance of tailoring treatment strategies to the specific subtypes in clinical practice.

### 3.2 Survival and Prognostic Factor Analyses Before PSM

**FIGURE 2:**
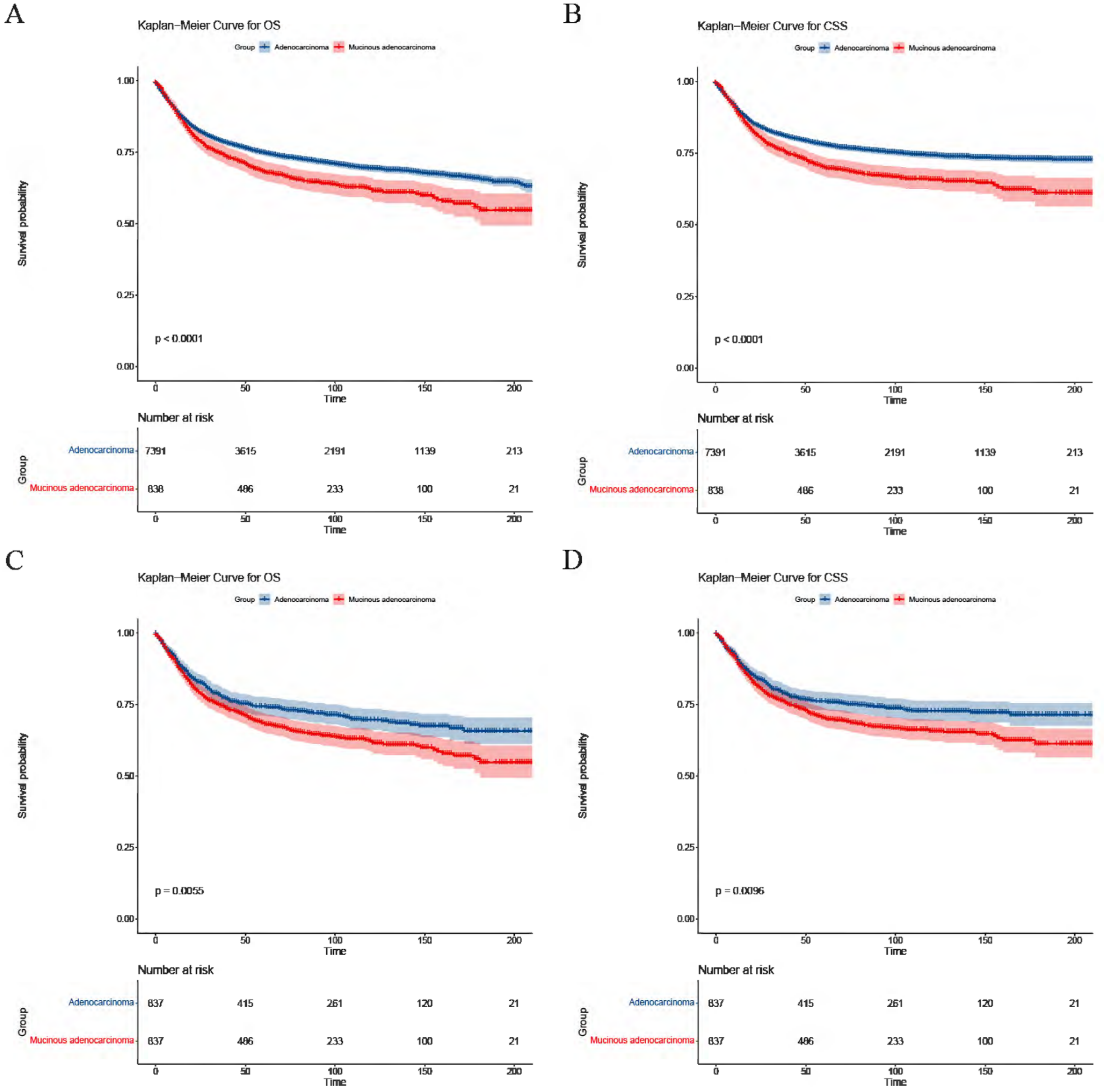
Kaplan–Meier survival analysis comparing adenocarcinoma and mucinous adenocarcinoma (MA) groups. (A) Overall survival (OS) before propensity score matching (PSM); (B) Cancer‐specific survival (CSS) before PSM; (C) OS after PSM; (D) CSS after PSM.

Survival analyses were performed between adenocarcinoma and MA groups. The results showed that there were significant differences in both OS (*p* < 0.0001) and CSS (*p* < 0.0001). The prognosis of adenocarcinoma was greatly better, with 1‐, 3‐, and 5‐year OS rates of 89.0% [95% confidence interval (95% CI): 88.3%–89.7%] versus 88.2% (95% CI: 86.0%–90.4%), 79.0% (95% CI: 78.0%–80.0%) versus 74.5% (95% CI: 71.5%–77.7%), and 74.9% (95% CI: 73.8%–76.0%) versus. 68.4% (95% CI: 65.1%–71.8%). Similarly, the 1,3 and 5‐year CSS rates were 90.4% (95% CI: 89.7%–91.1%) versus 89.3% (95% CI: 87.2%–91.5%), 81.5% (95% CI: 80.5%–82.4%) versus 76.2% (95% CI: 73.3%–79.3%), and 78.0% (95% CI: 77.0%–79.1%) versus 70.2% (95% CI: 67.0%–73.6%), respectively. The Kaplan–Meier Curve for OS and CSS before PSM was visualized in Figure 2A,B.

To identify the possible prognostic factors, we employed the univariate and multivariate Cox analyses in all included patients. The results were presented in Table 2. The poor prognosis of MA had been validated [hazard ratio (HR) = 1.52, 95% CI: 1.33–1.73, *p* < 0.001]. The findings of univariate Cox analyses revealed that race, age, origin, metropolitan or not, primary site, tumor size, grade, T, N, M, summary stage, combined stage, FIGO stage (2018), surgery, chemotherapy, and radiation were associated with both OS and CSS. However, in the multivariate Cox regression analyses, only race, age, origin, metropolitan or not, primary site, tumor size, grade, T, N, M, combined stage, FIGO stage (2018), surgery, and chemotherapy were identified as independent factors affecting patient survival. Receiving surgery (HR = 0.44, 95% CI: 0.39–0.50, *p* < 0.001) and chemotherapy (HR = 0.53, 95% CI: 0.47–0.59, *p* < 0.001) was associated with better prognosis.

**TABLE 2:** The univariate and multivariate cox regression analysis of OS and CSS before PSM.

Characteristic	OS				CSS			
	Univariate analysis		Multivariate analysis		Univariate analysis		Multivariate analysis	
	HR (95% CI)	*p*	HR (95% CI)	*p*	HR (95% CI)	*p*	HR (95% CI)	*p*
*Year of diagnosis*								
2004–2012	Reference				Reference			
2013–2021	0.93 (0.85–1.02)	0.105			0.92 (0.83–1.01)	0.077		
*Race*								
White	Reference		Reference		Reference		Reference	
Black	2.74 (2.42–3.1)	< 0.001	1.54 (1.36–1.76)	< 0.001	2.71 (2.36–3.11)	< 0.001	1.5 (1.3–1.73)	< 0.001
Unknown/other	1.07 (0.94–1.22)	0.278	1.04 (0.91–1.19)	0.5598	1.1 (0.96–1.27)	0.163	1.05 (0.91–1.21)	0.5231
*Age_group*								
< 50 years	Reference		Reference		Reference		Reference	
≥ 50 years	4.45 (4.06–4.87)	< 0.001	1.99 (1.8–2.19)	< 0.001	4.04 (3.66–4.46)	< 0.001	1.67 (1.5–1.86)	< 0.001
*Origin*								
Non‐Spanish‐Hispanic‐Latino	Reference		Reference		Reference		Reference	
Spanish‐Hispanic‐Latino	0.72 (0.64–0.8)	< 0.001	0.87 (0.78–0.98)	0.0246	0.72 (0.64–0.82)	< 0.001	0.86 (0.76–0.98)	0.0226
*Marital status*								
Married	Reference				Reference			
Others	1 (0.84–1.2)	0.999			1.06 (0.87–1.3)	0.572		
Unknown	1.02 (0.85–1.22)	0.825			1.1 (0.9–1.35)	0.357		
*Metropolitan or not*								
Metropolitan	Reference		Reference		Reference		Reference	
Others	0.7 (0.61–0.79)	< 0.001	0.79 (0.69–0.9)	0.0006	0.73 (0.63–0.84)	< 0.001	0.82 (0.71–0.96)	0.0103
*Income($)*								
< 60,000	Reference				Reference			
60,000–70,000	0.95 (0.81–1.12)	0.556			0.95 (0.79–1.14)	0.579		
70,000–80,000	1.01 (0.87–1.17)	0.915			1.06 (0.9–1.25)	0.466		
80,000–90,000	0.97 (0.83–1.14)	0.718			1.02 (0.85–1.21)	0.853		
90,000–100,000	0.98 (0.83–1.15)	0.792			1.02 (0.85–1.22)	0.846		
100,000+	0.95 (0.81–1.12)	0.972			1.07 (0.9–1.27)	0.457		
*Primary site*								
Endocervix	Reference		Reference		Reference		Reference	
Others	1.62 (1.49–1.77)	< 0.001	1.22 (1.11–1.33)	< 0.001	1.63 (1.48–1.79)	< 0.001	1.22 (1.11–1.35)	< 0.001
*Tumor size*								
≤ 20 mm	Reference		Reference		Reference		Reference	
20 mm‐40 mm	3.56 (2.92–4.32)	< 0.001	1.5 (1.22–1.84)	< 0.001	4.59 (3.61–5.82)	< 0.001	1.75 (1.36–2.25)	< 0.001
> 40 mm	10.21 (8.59–12.14)	< 0.001	1.83 (1.5–2.23)	< 0.001	14.01 (11.3–17.37)	< 0.001	2.22 (1.75–2.82)	< 0.001
Unknown	6.53 (5.51–7.75)	< 0.001	1.96 (1.62–2.37)	< 0.001	8.99 (7.26–11.13)	< 0.001	2.48 (1.96–3.13)	< 0.001
*Histological type*								
Adenocarcinoma	Reference		Reference		Reference		Reference	
Mucinous adenocarcinoma	1.29 (1.14–1.46)	< 0.001	1.52 (1.33–1.73)	< 0.001	1.38 (1.21–1.58)	< 0.001	1.59 (1.38–1.82)	< 0.001
*Grade*								
Well and moderately differentiated	Reference		Reference		Reference		Reference	
Poorly differentiated and undifferentiated	3.79 (3.41–4.22)	< 0.001	1.67 (1.49–1.86)	< 0.001	4.06 (3.62–4.56)	< 0.001	1.68 (1.49–1.9)	< 0.001
Unknown	2.15 (1.94–2.39)	< 0.001	1.09 (0.98–1.22)	0.1186	2.19 (1.95–2.45)	< 0.001	1.07 (0.95–1.2)	0.2859
*T*								
I–II	Reference		Reference		Reference		Reference	
III–IV	8.97 (8.15–9.88)	< 0.001	1.69 (1.51–1.89)	< 0.001	9.65 (8.7–10.7)	< 0.001	1.67 (1.48–1.89)	< 0.001
Unknown	5.11 (4.45–5.86)	< 0.001	1.79 (1.47–2.19)	< 0.001	5.27 (4.54–6.13)	< 0.001	1.78 (1.44–2.2)	< 0.001
*N*								
N0	Reference		Reference		Reference		Reference	
N1	4.95 (4.51–5.44)	< 0.001	1.17 (1.05–1.31)	0.0041	5.59 (5.05–6.18)	< 0.001	1.2 (1.07–1.35)	0.0019
Unknown	4.69 (4.1–5.36)	< 0.001	1.17 (0.95–1.42)	0.133	4.88 (4.2–5.65)	< 0.001	1.22 (0.98–1.51)	0.0688
*M*								
M0	Reference		Reference		Reference		Reference	
M1	9.32 (8.48–10.24)	< 0.001	1.47 (1.15–1.89)	0.0021	10.48 (9.48–11.59)	< 0.001	1.4 (1.09–1.81)	0.0094
Unknown	2.98 (2.33–3.82)	< 0.001	0.79 (0.58–1.07)	0.1232	3.15 (2.39–4.16)	< 0.001	0.97 (0.69–1.37)	0.8716
*Summary stage*								
I–II	Reference				Reference			
III–IV	11.09 (10.12–12.15)	< 0.001			12.6 (11.39–13.92)	< 0.001		
Unknown	3.8 (3.19–4.53)	< 0.001			3.69 (3.02–4.52)	< 0.001		
*Combined stage*								
Localized	Reference		Reference		Reference		Reference	
Regional	7.85 (6.94–8.87)	< 0.001	3.56 (2.92–4.34)	< 0.001	10.03 (8.65–11.63)	< 0.001	4.09 (3.24–5.15)	< 0.001
Distant	27.29 (24.01–31.01)	< 0.001	5.41 (3.97–7.38)	< 0.001	37.21 (31.99–43.29)	< 0.001	6.81 (4.85–9.56)	< 0.001
Unknown/others(in situ)	6.48 (5.19–8.11)	< 0.001	0.64 (0.45–0.92)	0.0155	7.11 (5.44–9.29)	< 0.001	0.65 (0.44–0.98)	0.0412
*FIGO stage (2018)*								
Before IIB	Reference		Reference		Reference		Reference	
IIB and after IIB	10.58 (9.52–11.75)	< 0.001	1.3 (1.08–1.56)	0.0063	13.76 (12.15–15.59)	< 0.001	1.51 (1.22–1.87)	< 0.001
Unknown	6.39 (5.39–7.57)	< 0.001	2.12 (1.56–2.88)	< 0.001	7.06 (5.78–8.62)	< 0.001	2.12 (1.49–3.01)	< 0.001
*Surgery*								
No	Reference		Reference		Reference		Reference	
Yes	0.13 (0.12–0.14)	< 0.001	0.44 (0.39–0.5)	< 0.001	0.13 (0.12–0.14)	< 0.001	0.49 (0.43–0.55)	< 0.001
*Chemotherapy*								
No/unknown	Reference		Reference		Reference		Reference	
Yes	3.43 (3.14–3.74)	< 0.001	0.53 (0.47–0.59)	< 0.001	3.9 (3.54–4.31)	< 0.001	0.55 (0.49–0.62)	< 0.001
*Radiation*								
No/unknown	Reference				Reference			
Yes	2.92 (2.68–3.19)	< 0.001			3.06 (2.78–3.38)	< 0.001		

Abbreviations: 95% CI, 95% confidence interval; CSS, cancer‐specific survival; FIGO, International Federation of Gynecology and Obstetrics; HR, hazard ratio; OS, overall survival; PSM, Propensity Score Matching.

**FIGURE 3:**
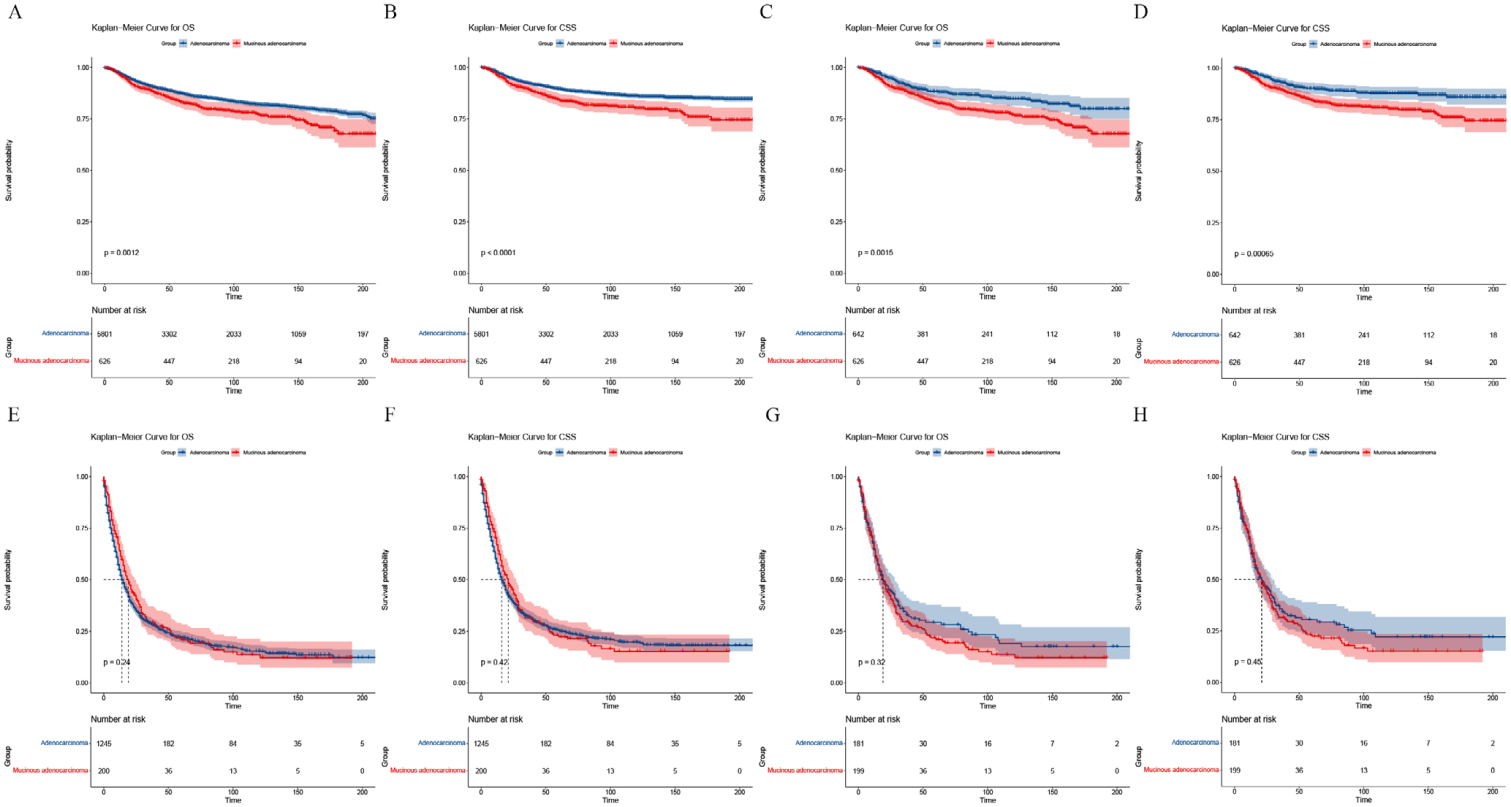
Stage‐stratified Kaplan–Meier survival analysis. (A) OS between adenocarcinoma group and MA group of patients in I‐II stages before PSM; (B) CSS between adenocarcinoma group and MA group of patients in I‐II stages before PSM; (C) OS between adenocarcinoma group and MA group of patients in III‐IV stages before PSM; (D) CSS between adenocarcinoma group and MA group of patients in III‐IV stages before PSM; (E) OS between adenocarcinoma group and MA group of patients in I‐II stages after PSM; (F) CSS between adenocarcinoma group and MA group of patients in I‐II stages after PSM; (G) OS between adenocarcinoma group and MA group of patients in III‐IV stages after PSM; (H) CSS between adenocarcinoma group and MA group of patients in III‐IV stages after PSM.

Furthermore, we conducted survival analyses on subgroups of different summary stages (I‐II stage group and III‐IV stage group). The results were shown in Figure 3A–D. In all different subgroups, the prognosis of MA was worse.

### 3.3 Survival and Prognostic Factor Analysis After PSM

After PSM, which balanced the difference between adenocarcinoma and MA groups, a total of 1674 patients were selected (Table 1). The jittering with stripplot showed the results of PSM was visualized in Figure S1. Subsequently, we conducted survival analyses, revealing race, age, primary site, tumor size, histological type, grade, T, N, summary stage, combined stage, surgery, and chemotherapy were associated with OS. However, year of diagnosis, race, age, primary site, tumor size, histological type, grade, T, N, combined stage, and surgery were defined as independent prognostic factors of CSS. The results confirmed that surgery was a protective factor for prognosis (HR = 0.49, 95% CI: 0.40–0.62, *p* < 0.001). The details were presented in Table 3.

**TABLE 3:** The univariate and multivariate cox regression analysis of OS and CSS after PSM.

Characteristic	OS				CSS			
	Univariate analysis		Multivariate analysis		Univariate analysis		Multivariate analysis	
	HR (95% CI)	*p*	HR (95% CI)	*p*	HR (95% CI)	*p*	HR (95% CI)	*p*
*Year of diagnosis*								
2004–2012	Reference				Reference		Reference	
2013–2021	0.84 (0.7–1.01)	0.06			0.79 (0.65–0.96)	0.018	0.73 (0.59–0.89)	0.0023
*Race*								
White	Reference		Reference		Reference		Reference	
Black	2.01 (1.47–2.76)	< 0.001	1.76 (1.27–2.43)	0.007	1.94 (1.38–2.73)	< 0.001	1.68 (1.18–2.39)	0.0039
Unknown/other	1.36 (1.08–1.72)	0.01	1.33 (1.05–1.69)	0.0176	1.36 (1.06–1.74)	0.015	1.37 (1.07–1.77)	0.0141
*Age_group*								
< 50	Reference		Reference		Reference		Reference	
≥ 50	3.21 (2.67–3.87)	< 0.001	1.8 (1.49–2.19)	< 0.001	2.96 (2.43–3.59)	< 0.001	1.62 (1.32–1.99)	< 0.001
*Origin*								
Non‐Spanish‐Hispanic‐Latino	Reference				Reference			
Spanish‐Hispanic‐Latino	0.97 (0.78–1.22)	0.818			0.97 (0.76–1.22)	0.782		
*Marital statue*								
Married	Reference				Reference			
Others	1.06 (0.73–1.54)	0.75			1.07 (0.72–1.58)	0.746		
Unknown	1.03 (0.71–1.49)	0.876			1.06 (0.71–1.57)	0.777		
*Metropolitan or not*								
Metropolitan	Reference				Reference			
Others	0.96 (0.71–1.31)	0.803			1.05 (0.75–1.47)	0.786		
*Income($)*								
< 60,000	Reference				Reference			
60,000–70,000	0.83 (0.58–1.18)	0.296			0.85 (0.58–1.25)	0.418		
70,000–80,000	1.08 (0.79–1.46)	0.64			1.1 (0.79–1.53)	0.562		
80,000–90,000	0.93 (0.66–1.3)	0.662			0.91 (0.64–1.3)	0.598		
90,000–100,000	1.01 (0.71–1.45)	0.938			1.03 (0.7–1.5)	0.895		
100,000+	0.95 (0.68–1.32)	0.747			0.95 (0.67–1.36)	0.784		
*Primary site*								
Endocervix	Reference		Reference		Reference		Reference	
Others	1.67 (1.4–1.99)	< 0.001	1.35 (1.12–1.63)	0.0018	1.69 (1.4–2.04)	< 0.001	1.37 (1.12–1.68)	0.0021
*Tumor size*								
≤ 20 mm	Reference		Reference		Reference		Reference	
20 mm–40 mm	2.76 (1.87–4.07)	< 0.001	1.46 (0.97–2.19)	0.0696	3.48 (2.19–5.52)	< 0.001	1.69 (1.04–2.72)	0.0327
> 40 mm	8.19 (5.79–11.58)	< 0.001	1.96 (1.33–2.9)	< 0.001	11.4 (7.51–17.31)	< 0.001	2.54 (1.61–4)	< 0.001
Unknown	7.11 (5–10.11)	< 0.001	2.29 (1.56–3.38)	< 0.001	9.91 (6.5–15.13)	< 0.001	2.88 (1.83–4.55)	< 0.001
*Histological type*								
Adenocarcinoma	Reference		Reference		Reference		Reference	
Mucinous adenocarcinoma	1.29 (1.08–1.54)	0.006	1.4 (1.16–1.68)	< 0.001	1.28 (1.06–1.55)	0.01	1.42 (1.16–1.72)	< 0.001
*Grade*								
Well and moderately differentiated	Reference		Reference		Reference		Reference	
Poorly differentiated and undifferentiated	2.5 (2–3.13)	< 0.001	1.42 (1.12–1.8)	0.0033	2.71 (2.14–3.43)	< 0.001	1.47 (1.15–1.88)	0.0023
Unknown	3.13 (2.54–3.87)	< 0.001	1.19 (0.95–1.5)	0.1374	3.23 (2.58–4.03)	< 0.001	1.23 (0.97–1.58)	0.092
*T*								
I–II	Reference		Reference		Reference		Reference	
III–IV	7.97 (6.57–9.67)	< 0.001	1.96 (1.56–2.46)	< 0.001	8.48 (6.93–10.39)	< 0.001	1.94 (1.54–2.46)	< 0.001
Unknown	7.2 (4.81–10.78)	< 0.001	1.2 (0.71–2.02)	0.4873	7.68 (5.04–11.7)	< 0.001	1.19 (0.69–2.06)	0.5268
*N*								
N0	Reference		Reference		Reference		Reference	
N1	3.83 (3.17–4.63)	< 0.001	1.25 (1.01–1.55)	0.0386	4 (3.28–4.9)	< 0.001	1.22 (0.97–1.52)	0.0856
Unknown	6.67 (4.94–9)	< 0.001	2.03 (1.41–2.93)	< 0.001	6.82 (4.97–9.35)	< 0.001	1.92 (1.3–2.83)	0.001
*M*								
M0	Reference		Reference		Reference		Reference	
M1	7.59 (6.28–9.17)	< 0.001	1.48 (0.92–2.37)	0.1046	7.96 (6.52–9.72)	< 0.001	1.33 (0.82–2.14)	0.2451
Unknown	5.46 (3.25–9.18)	< 0.001	1.8 (0.93–3.5)	0.0804	5.95 (3.47–10.19)	< 0.001	1.87 (0.94–3.71)	0.075
*Summary stage*								
I–II	Reference				Reference			
III–IV	9.17 (7.6–11.05)	< 0.001			10.06 (8.24–12.29)	< 0.001		
Unknown	6.31 (3.79–10.5)	< 0.001			6.72 (3.89–11.6)	< 0.001		
*Combined stage*								
Localized	Reference		Reference		Reference		Reference	
Regional	7.1 (5.39–9.35)	< 0.001	3.88 (2.75–5.49)	< 0.001	9.48 (6.84–13.12)	< 0.001	4.9 (3.3–7.28)	< 0.001
Distant	23.71 (17.91–31.4)	< 0.001	7.42 (4.27–12.9)	< 0.001	31.96 (23–44.41)	< 0.001	10.25 (5.71–18.4)	< 0.001
Unknown/others(in situ)	7.46 (3.01–18.5)	< 0.001	0.72 (0.24–2.18)	0.5613	11.2 (4.45–28.23)	< 0.001	1.06 (0.34–3.32)	0.9192
*FIGO stage (2018)*								
Before IIB	Reference				Reference			
IIB and after IIB	9.24 (7.34–11.62)	< 0.001			11.36 (8.75–14.76)	< 0.001		
Unknown	9.34 (6.11–14.27)	< 0.001			11.09 (7.01–17.56)	< 0.001		
*Surgery*								
No	Reference		Reference		Reference		Reference	
Yes	0.16 (0.14–0.19)	< 0.001	0.49 (0.4–0.62)	< 0.001	0.16 (0.13–0.19)	< 0.001	0.52 (0.41–0.66)	< 0.001
*Chemotherapy*								
No/unknown	Reference		Reference		Reference		Reference	
Yes	3.78 (3.1–4.61)	< 0.001	0.64 (0.5–0.83)	< 0.001	4.52 (3.62–5.64)	< 0.001	0.76 (0.58–1.01)	0.0581
*Radiation*								
No/unkown	Reference		Reference		Reference		Reference	
Yes	2.72 (2.24–3.29)	< 0.001	1.19 (0.94–1.52)	0.1526	2.85 (2.32–3.5)	< 0.001	1.07 (0.83–1.39)	0.6075

Abbreviations: 95% CI, 95% confidence interval; CSS, cancer‐specific survival; FIGO, International Federation of Gynecology and Obstetrics; HR, hazard ratio; OS, overall survival; PSM, Propensity Score Matching.

Compare to the findings of PSM, there was no difference between adenocarcinoma and MA groups in the survival analyses on different summary stage subgroups (I‐II stage group and III‐IV stage group). The Kaplan–Meier Curve for OS and CSS was visualized in Figure 3E–H.

### 3.4 Development and Validation of Risk Prediction Model for MA

**FIGURE 4:**
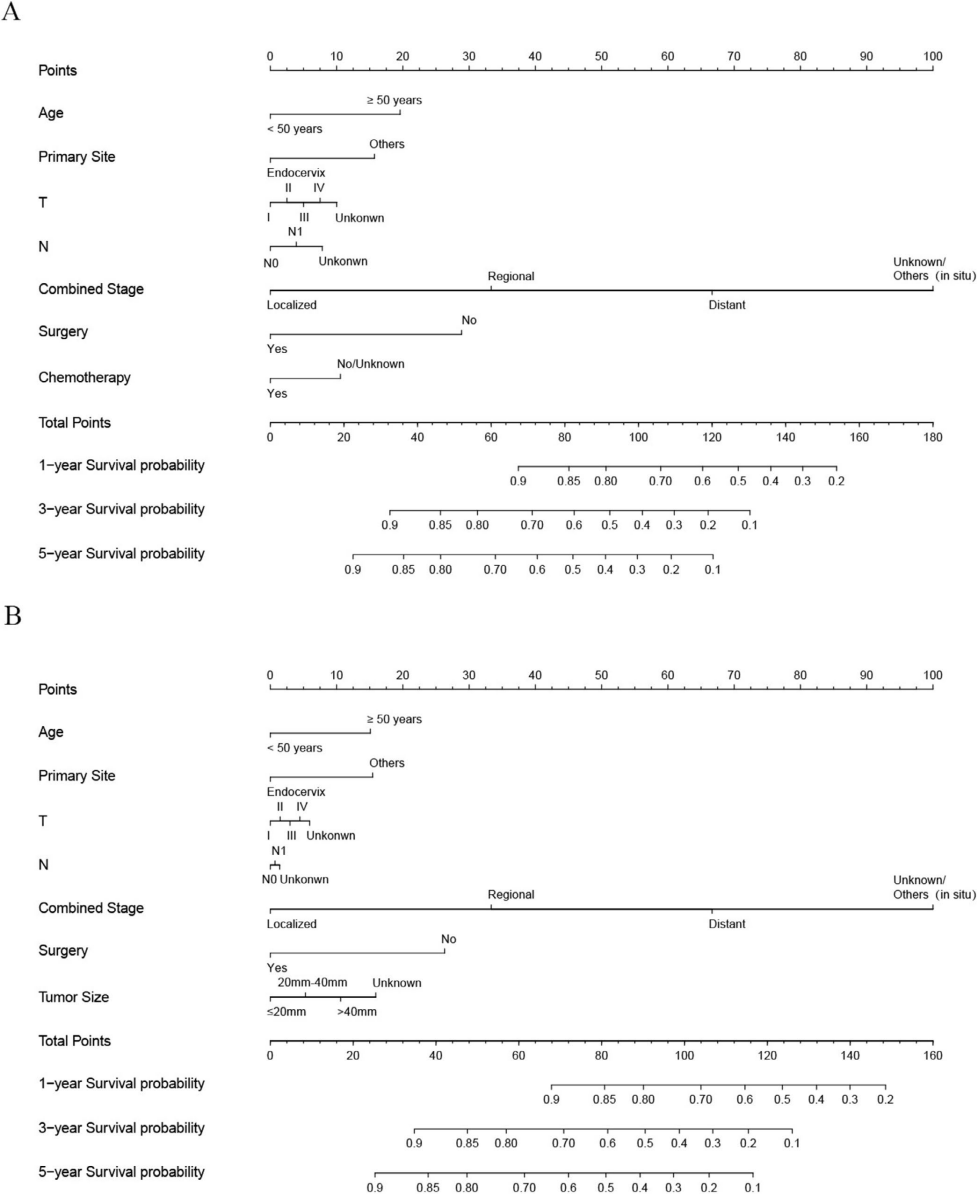
Nomogram prognostic models for MA patients: (A) OS prediction; (B) CSS prediction.

**FIGURE 5:**
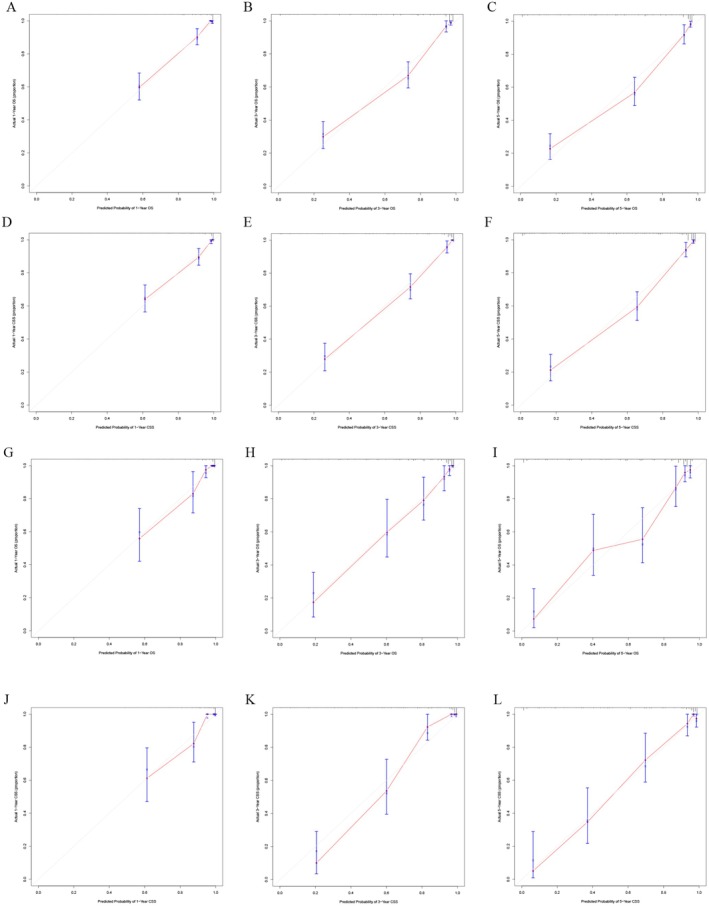
Calibration curves for MA prognostic models. Training cohort: 1−/3−/5‐year OS (A–C) and CSS (D–F); Validation cohort: 1−/3−/5‐year OS (G–I) and CSS (J–L). Dashed diagonal lines represent ideal predictions.

**FIGURE 6:**
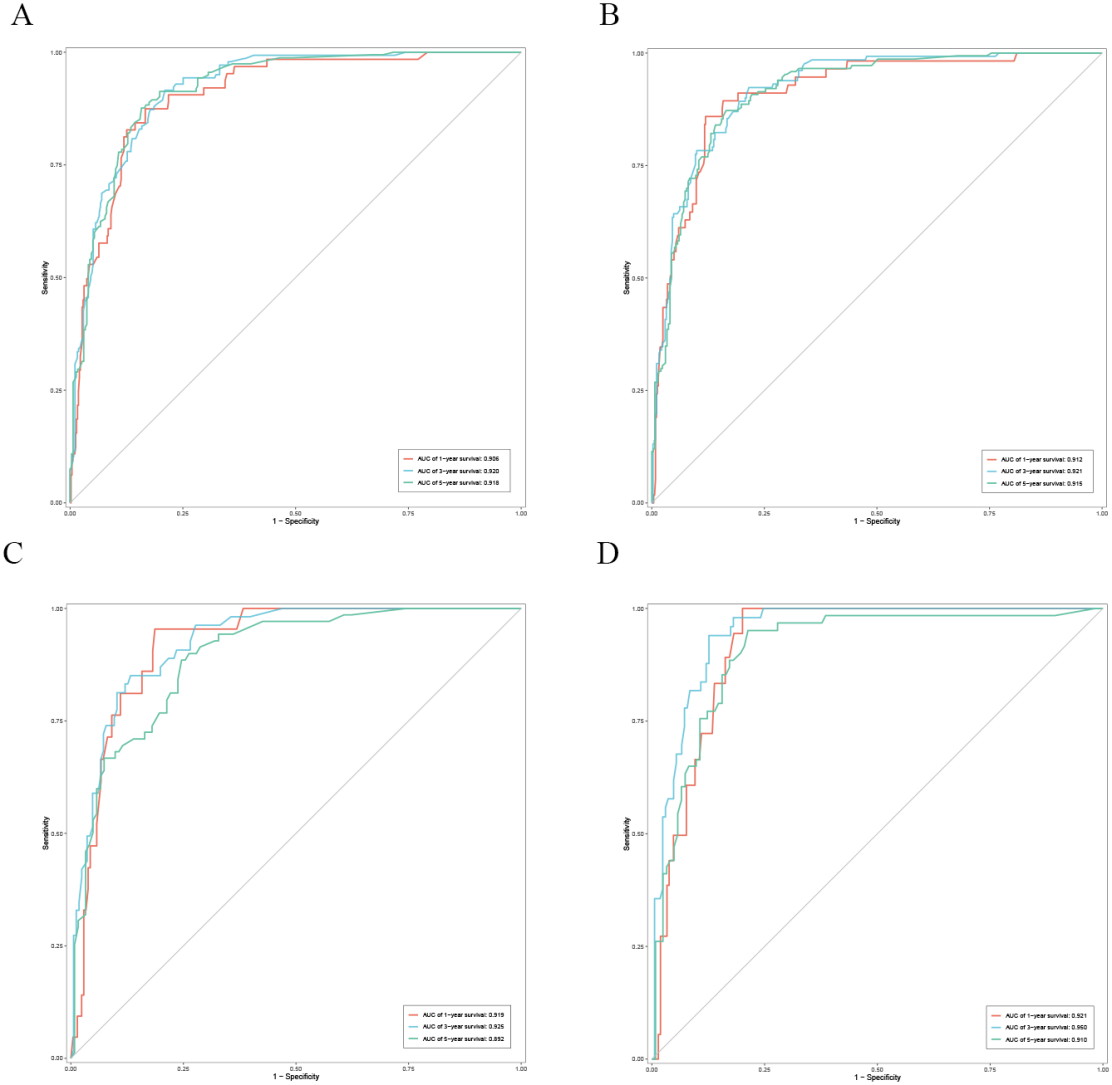
Time‐dependent receiver operating characteristic (ROC) curves. Training cohort: OS (A) and CSS (B); Validation cohort: OS (C) and CSS (D). AUC, area under the curve.

**FIGURE 7:**
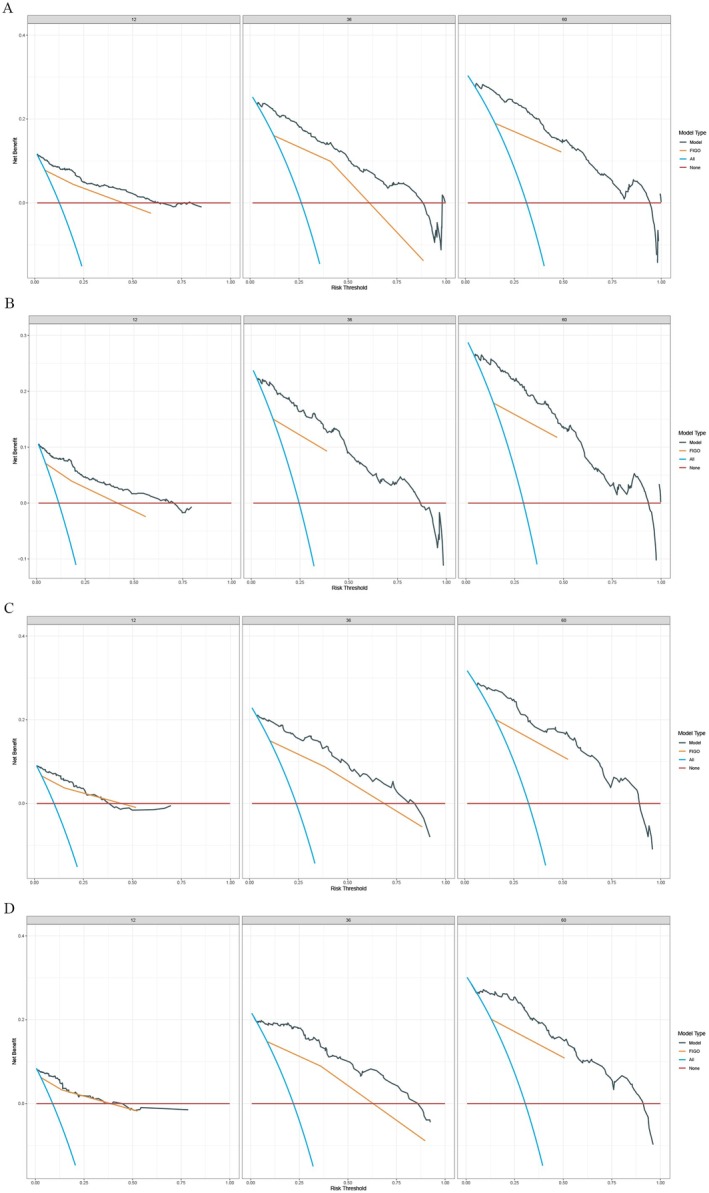
Decision curve analysis (DCA) evaluating clinical utility. Training cohort: OS (A) and CSS (B); Validation cohort: OS (C) and CSS (D).

The training set included 588 patients with MA and the validation set had 250 patients. There was no difference in the clinicopathological features of the two groups. The baseline was shown in Table 4. By performing univariate and multivariate Cox analyses on the training set, we selected seven variables including age, primary site, T, N, combined stage, surgery, and chemotherapy to create the nomograms for predicting OS (Figure 4A). While seven variables including age, primary site, tumor size, T, N, combined stage, and surgery were used for CSS (Figure 4B). The calibration curve of 1, 3, and 5‐year for OS visualized the consistency between the predicted and observed probabilities of the model in both training and validation set (Figure 5A–F). The results of CSS were similar to the OS (Figure 5G–L). Subsequently, time‐ROC analyses were employed to assess the predictive accuracy of the model at 3 different time points (1 year, 3 years, and 5 years). The models of OS showed pretty predictive performance, with the AUC values of 0.906 for 1‐year, 0.920 for 3 years, and 0.918 for 5 years in the training set (Figure 6A), while in the validation set, they were 0.919, 0.925, and 0.892 (Figure 6C). Similar to the OS, the results of CSS showed satisfactory outcomes. The AUC values for three time points were 0.912, 0.921, and 0.915, respectively, in the training set (Figure 6B), while in the validation set, they were 0.921, 0.950, and 0.910 (Figure 6D). The DCA curves further demonstrated that predictive models for OS had excellent clinical benefits at different time intervals, both in the training and validation sets (Figure 7A,B). The validation of the CSS predictive model had the same result (Figure 7C,D).

**TABLE 4:** Comparison of baseline characteristics between the training set and the validation set in patients of mucinous adenocarcinoma.

Characteristic	Overall (*N* = 838)	Training set (*N* = 588)	Validation set (*N* = 250)	*p*
*Year of diagnosis*				
2004–2012	376 (44.9%)	262 (44.6%)	114 (45.6%)	0.82
2013–2021	462 (55.1%)	326 (55.4%)	136 (54.4%)	
*Race*				
White	650 (77.6%)	460 (78.2%)	190 (76.0%)	0.708
Black	48 (5.73%)	34 (5.78%)	14 (5.60%)	
Unknown/other	140 (16.7%)	94 (16.0%)	46 (18.4%)	
*Age_group*				
< 50	486 (58.0%)	349 (59.4%)	137 (54.8%)	0.251
≥ 50	352 (42.0%)	239 (40.6%)	113 (45.2%)	
*Origin*				
Non‐Spanish‐Hispanic‐Latino	645 (77.0%)	448 (76.2%)	197 (78.8%)	0.422
Spanish‐Hispanic‐Latino	193 (23.0%)	140 (23.8%)	53 (21.2%)	
*Marital statue*				
Married	61 (7.28%)	39 (6.63%)	22 (8.80%)	0.416
Others	402 (48.0%)	289 (49.1%)	113 (45.2%)	
Unknown	375 (44.7%)	260 (44.2%)	115 (46.0%)	
*Metropolitan or not*				
Metropolitan	76 (9.07%)	53 (9.01%)	23 (9.20%)	1
Others	762 (90.9%)	535 (91.0%)	227 (90.8%)	
*Income($)*				
< 60,000	100 (11.9%)	68 (11.6%)	32 (12.8%)	0.276
60,000–70,000	119 (14.2%)	78 (13.3%)	41 (16.4%)	
70,000–80,000	204 (24.3%)	142 (24.1%)	62 (24.8%)	
80,000–90,000	145 (17.3%)	113 (19.2%)	32 (12.8%)	
90,000–100,000	110 (13.1%)	79 (13.4%)	31 (12.4%)	
100,000+	160 (19.1%)	108 (18.4%)	52 (20.8%)	
*Primary site*				
Endocervix	465 (55.5%)	335 (57.0%)	130 (52.0%)	0.197
Others	373 (44.5%)	253 (43.0%)	120 (48.0%)	
*Tumor size*				
≤ 20 mm	230 (27.4%)	166 (28.2%)	64 (25.6%)	0.789
20 mm–40 mm	185 (22.1%)	125 (21.3%)	60 (24.0%)	
> 40 mm	220 (26.3%)	155 (26.4%)	65 (26.0%)	
Unknown	203 (24.2%)	142 (24.1%)	61 (24.4%)	
*Grade*				
Well and moderately differentiated	539 (64.3%)	380 (64.6%)	159 (63.6%)	0.768
Poorly differentiated and undifferentiated	123 (14.7%)	83 (14.1%)	40 (16.0%)	
Unknown	176 (21.0%)	125 (21.3%)	51 (20.4%)	
*T*				
I	471 (56.2%)	344 (58.5%)	127 (50.8%)	0.105
II	222 (26.5%)	148 (25.2%)	74 (29.6%)	
III	88 (10.5%)	56 (9.52%)	32 (12.8%)	
IV	39 (4.65%)	30 (5.10%)	9 (3.60%)	
Unknown	18 (2.15%)	10 (1.70%)	8 (3.20%)	
*N*				
N0	618 (73.7%)	430 (73.1%)	188 (75.2%)	0.353
N1	182 (21.7%)	134 (22.8%)	48 (19.2%)	
Unknown	38 (4.53%)	24 (4.08%)	14 (5.60%)	
*M*				
M0	699 (83.4%)	492 (83.7%)	207 (82.8%)	0.818
M1	129 (15.4%)	90 (15.3%)	39 (15.6%)	
Unknown	10 (1.19%)	6 (1.02%)	4 (1.60%)	
*Summary stage*				
I–II	626 (74.7%)	443 (75.3%)	183 (73.2%)	0.316
III–IV	200 (23.9%)	139 (23.6%)	61 (24.4%)	
Unknown	12 (1.43%)	6 (1.02%)	6 (2.40%)	
*Combined stage*				
Localized	425 (50.7%)	300 (51.0%)	125 (50.0%)	0.27
Regional	263 (31.4%)	184 (31.3%)	79 (31.6%)	
Distant	144 (17.2%)	102 (17.3%)	42 (16.8%)	
Unknown/others(in situ)	6 (0.72%)	2 (0.34%)	4 (1.60%)	
*FIGO stage (2018)*				
Before IIB	479 (57.2%)	338 (57.5%)	141 (56.4%)	0.717
IIB and after IIB	335 (40.0%)	235 (40.0%)	100 (40.0%)	
Unknown	24 (2.86%)	15 (2.55%)	9 (3.60%)	
*Surgery*				
No	190 (22.7%)	129 (21.9%)	61 (24.4%)	0.471
Yes	648 (77.3%)	459 (78.1%)	189 (75.6%)	
*Chemotherapy*				
No/unknown	438 (52.3%)	307 (52.2%)	131 (52.4%)	1
Yes	400 (47.7%)	281 (47.8%)	119 (47.6%)	
*Radiation*				
No/unknown	423 (50.5%)	294 (50.0%)	129 (51.6%)	0.706
Yes	415 (49.5%)	294 (50.0%)	121 (48.4%)	

Abbreviation: FIGO, International Federation of Gynecology and Obstetrics.

We collected clinical data from 65 patients with MA at Fujian Provincial Cancer Hospital (Table 5). The baseline characteristics of the patients are detailed in Table S1. Calibration curve analysis demonstrated that our predictive model had a good fit and predictive accuracy for both 3‐year and 5‐year survival rates (Figure S2). External validation using time‐dependent ROC curves showed AUC values of 0.928 and 0.853 for 3‐year and 5‐year survival rates, respectively, indicating high predictive accuracy. Further decision curve analysis (DCA) also suggested that the predictive model has good practicality and potential value for clinical application (Figure S3).

**TABLE 5:** The univariate and multivariate cox regression analysis of OS and CSS in the training set.

Characteristic	OS				CSS			
	Univariate analysis		Multivariate analysis		Univariate analysis		Multivariate analysis	
	HR (95% CI)	*p*	HR (95% CI)	*p*	HR (95% CI)	*p*	HR (95% CI)	*p*
*Year of diagnosis*								
2004–2012	Reference				Reference			
2013–2021	0.88 (0.66–1.18)	0.393			0.79 (0.59–1.08)	0.137		
*Race*								
White	Reference				Reference			
Black	1.47 (0.85–2.54)	0.171			1.41 (0.78–2.55)	0.252		
Unknown/other	1.22 (0.84–1.78)	0.3			1.33 (0.91–1.96)	0.144		
*Age_group*								
< 50 years	Reference		Reference		Reference		Reference	
≥ 50 years	3.17 (2.37–4.23)	< 0.001	1.76 (1.3–2.38)	< 0.001	2.86 (2.12–3.87)	< 0.001	1.51 (1.1–2.08)	0.0103
*Origin*								
Non‐Spanish‐Hispanic‐Latino	Reference				Reference			
Spanish‐Hispanic‐Latino	1.08 (0.78–1.51)	0.643			1.06 (0.75–1.51)	0.742		
*Marital status*								
Married	Reference				Reference			
Others	0.75 (0.43–1.32)	0.317			0.85 (0.45–1.6)	0.617		
Unknown	0.94 (0.53–1.64)	0.822			1.11 (0.6–2.09)	0.735		
*Metropolitan or not*								
Metropolitan	Reference				Reference			
Others	0.94 (0.58–1.5)	0.788			0.94 (0.57–1.56)	0.819		
*Income($)*								
< 60,000	Reference				Reference			
60,000–70,000	0.74 (0.4–1.34)	0.317			0.85 (0.45–1.61)	0.625		
70,000–80,000	1.1 (0.68–1.79)	0.701			1.27 (0.75–2.16)	0.366		
80,000–90,000	0.81 (0.48–1.37)	0.425			0.89 (0.51–1.58)	0.697		
90,000–100,000	1.03 (0.59–1.79)	0.913			1.02 (0.56–1.87)	0.947		
100,000+	0.92 (0.55–1.56)	0.759			0.96 (0.54–1.69)	0.878		
*Primary site*								
Endocervix	Reference		Reference		Reference		Reference	
Others	1.59 (1.2–2.1)	< 0.001	1.49 (1.1–2.01)	0.0097	1.63 (1.21–2.19)	< 0.001	1.57 (1.13–2.16)	0.0065
*Tumor size*								
≤ 20 mm	Reference				Reference		Reference	
20 mm–40 mm	3.6 (1.93–6.73)	< 0.001			4.71 (2.31–9.62)	< 0.001	2.37 (1.13–4.97)	0.0228
> 40 mm	9.65 (5.47–17.02)	< 0.001			12.32 (6.36–23.86)	< 0.001	2.56 (1.24–5.26)	0.0108
Unknown	7.31 (4.1–13.02)	< 0.001			9.22 (4.71–18.02)	< 0.001	2.37 (1.14–4.95)	0.0215
*Grade*								
Well and moderately differentiated	Reference				Reference			
Poorly differentiated and undifferentiated	2.1 (1.44–3.06)	< 0.001			2.2 (1.48–3.27)	< 0.001		
Unknown	3.2 (2.32–4.4)	< 0.001			3.26 (2.33–4.57)	< 0.001		
*T*								
I	Reference		Reference		Reference		Reference	
II	3.45 (2.4–4.95)	< 0.001	1.75 (1.19–2.57)	0.0048	3.85 (2.61–5.68)	< 0.001	1.8 (1.19–2.73)	0.0055
III	9.8 (6.52–14.73)	< 0.001	2.67 (1.71–4.16)	< 0.001	10.93 (7.09–16.86)	< 0.001	2.77 (1.7–4.49)	< 0.001
IV	16.95 (10.23–28.1)	< 0.001	2.16 (1.22–3.84)	0.0087	19.42 (11.45–32.93)	< 0.001	2.38 (1.28–4.43)	0.006
Unknown	9.33 (4.42–19.7)	< 0.001	1.07 (0.45–2.55)	0.8744	9.8 (4.4–21.84)	< 0.001	1.15 (0.45–2.93)	0.7623
*N*								
N0	Reference		Reference		Reference		Reference	
N1	4.14 (3.08–5.57)	< 0.001	1.51 (1.1–2.08)	0.0119	4.14 (3.04–5.64)	< 0.001	1.46 (1.04–2.05)	0.0268
Unknown	4.52 (2.62–7.81)	< 0.001	1.74 (0.94–3.24)	0.0803	3.59 (1.91–6.73)	< 0.001	1.3 (0.63–2.7)	0.4813
*M*								
M0	Reference				Reference			
M1	6.92 (5.12–9.36)	< 0.001			6.82 (4.97–9.37)	< 0.001		
Unknown	2.25 (0.71–7.06)	0.167			6.82 (4.97–9.37)	0.472		
*Summary Stage*								
I–II	Reference				Reference			
III–IV	8.35 (6.24–11.18)	< 0.001			8.52 (6.26–11.58)	< 0.001		
Unknown	2.99 (0.95–9.47)	0.062			2.27 (0.56–9.26)	0.252		
*Combined stage*								
Localized	Reference		Reference		Reference		Reference	
Regional	10.61 (6.6–17.05)	< 0.001	5.59 (3.2–9.77)	< 0.001	11.73 (6.96–19.77)	< 0.001	4.81 (2.59–8.93)	< 0.001
Distant	31.34 (19.2–51.15)	< 0.001	16.96 (9.24–31.13)	< 0.001	34.74 (20.3–59.42)	< 0.001	15.26 (7.77–29.98)	< 0.001
Unknown/others(in situ)	6.33 (0.85–47.14)	0.072	2.82 (0.31–25.49)	0.3564	7.92 (1.05–59.66)	0.045	3.62 (0.38–34.72)	0.2653
*FIGO stage (2018)*								
Before IIB	Reference				Reference			
IIB and after IIB	11.49 (7.9–16.71)	< 0.001			12.1 (8.09–18.11)	< 0.001		
Unknown	6.32 (2.8–14.27)	< 0.001			6.28 (2.61–15.14)	< 0.001		
*Surgery*								
No	Reference		Reference		Reference		Reference	
Yes	0.17 (0.13–0.23)	< 0.001	0.41 (0.3–0.56)	< 0.001	0.17 (0.13–0.24)	< 0.001	0.47 (0.33–0.66)	< 0.001
*Chemotherapy*								
No/unknown	Reference		Reference		Reference		Reference	
Yes	3.87 (2.83–5.31)	< 0.001	0.68 (0.46–1)	0.0477	4.53 (3.2–6.39)	< 0.001	0.72 (0.47–1.09)	0.1192
*Radiation*								
No/unknown	Reference				Reference			
Yes	2.9 (2.13–3.95)	< 0.001			3.06 (2.2–4.25)	< 0.001		

Abbreviations: 95% CI, 95% confidence interval; CSS, cancer‐specific survival; FIGO, International Federation of Gynecology and Obstetrics; HR, hazard ratio; OS, overall survival; PSM, Propensity Score Matching.

## 4 Discussion

MA of the cervix exhibits significant molecular and histological differences compared to typical cervical adenocarcinoma, which may explain its poorer prognosis [8]. From a histological perspective, MA is characterized by the production of large amounts of mucus, with tumor cells often forming glandular structures accompanied by notable extracellular mucin accumulation. This mucus secretion may contribute to the enhanced invasiveness of tumor cells both locally and in distant metastases. In contrast, typical adenocarcinoma usually displays more typical glandular structures with less mucus secretion and relatively lower cellular atypia [16]. Cervical MA is rare and has a poor prognosis, with its pathogenesis and clinical features lacking clear distinguishing characteristics. Surgical indications may be somewhat more lenient, and early diagnosis and treatment can significantly improve patient outcomes [17]. In addition to clinicopathological factors, emerging molecular evidence may further explain the survival disparity between MA and usual‐type adenocarcinoma. Recent classifications such as the 2020 World Health Organization (WHO) Classification of cervical tumors distinguish HPV‐associated (HPVA) and HPV‐independent (HPVI) subtypes [18]. MA, particularly gastric‐type adenocarcinoma (a common HPVI subtype), frequently harbors mutations in signaling pathways such as KRAS/TP53 and shows HER2 amplification, which are associated with resistance to conventional therapies and poor prognosis [19, 20, 21]. These features may contribute to its aggressive biological behavior, such as increased metastatic potential and reduced chemosensitivity. Integrating molecular markers (e.g., KRAS status) with clinical parameters could enhance risk stratification and guide targeted therapies (e.g., HER2 inhibitors) in future studies. Currently, there is a lack of large‐scale studies specifically focused on cervical MA as a distinct entity, and research on its treatment is particularly scarce. Therefore, further discussions and research are needed to enhance the diagnosis, treatment, and prognosis of cervical MA, with the goal of improving patient survival rates. In this investigation, we used data from the SEER database spanning from 2004 to 2021, further revealing the recognizable clinicopathological features and distinguishable survival outcomes between MA and adenocarcinoma.

Our results indicate that compared to the adenocarcinoma, MA occurred at older ages and exhibited larger tumor sizes and more advanced TNM stages. This is consistent with the studies by Xin Tian et al. [14] and Lucie Bonin et al. [22]. This may be attributed to the nonspecific clinical presentation, the secretive primary lesion, and the biologically aggressive phenotype of MA. In terms of survival outcomes, the 1, 3, and 5‐year OS rates and CSS rates of MA were poorer than those of adenocarcinoma in all group analyses. Studies by Tsukasa Saida et al. [23] reported that the 5‐year disease‐free survival rate of MA was much lower than that of adenocarcinoma. These findings collectively indicated a worse prognosis for MA. Our multivariate Cox regression analyses further confirmed that MA is associated with a poorer prognosis. Moreover, older age, larger tumor size, non‐endocervix primary site, poorer differentiation grade, more advanced grade, and radiation were recognized as independent factors associated with lower OS and CSS. However, surgery and chemotherapy were found to be favorable factors for survival. Even the effect of chemotherapy on CSS was not statistically significant (HR = 0.76, 95% CI: 0.58–1.01, *p* = 0.0581). There was no evidence that radiation had an impact on prognosis. The result was similar to a study by Chen et al. [24], which included 194 patients with stage IIB or higher cervical adenocarcinoma or adenosquamous carcinoma after radical radiotherapy or simultaneous radiochemotherapy. The study found that the 5‐year survival rate of adenocarcinoma was lower than that of concurrent squamous cervical carcinoma, and their 5‐year local recurrence rate was higher than that of concurrent squamous carcinoma.

At present, large cohort studies on prognostic factors of cervical MA are still lacking. Therefore, we built nomograms that forecast its 1,3,5 years OS and CSS. Five clinical factors including age, primary site, T, N, and combined stage were defined as independent risk factors for prognosis, while surgery and chemotherapy were independent protective factors to estimate OS for MA. For CSS, tumor size was considered an independent risk factor, while chemotherapy was excluded. The study by Yiping Hao et al. [25] identified age, stage, and tumor size as independent prognostic factors for MA. Their study included only 3 variables to construct a nomogram and lacked other important clinicopathological factors. Through univariate and multivariate Cox analyses, we finally screened 5 variables to build risk prediction models. Tumor stage is essential in predicting the prognosis of MA, with later tumor staging implying a higher probability of metastasis and a poorer prognosis [7]. Therefore, it is important for early recognition of MA. Currently, the commonly used diagnostic markers are CEA, CA199, etc. [26]. However, these indicators have only proved to be applicable to cervical adenocarcinoma, and their universality for MA has yet to be investigated, and we should pay more attention to this aspect in subsequent studies. This is useful for clinicians to identify MA early and improve its prognosis.

The current treatment for MA is based on surgery, which is also consistent with our study that surgery was a protective factor. For locally advanced MA, chemotherapy is usually combined with radiotherapy (simultaneous radiotherapy and chemotherapy), which is considered one of the standard treatments. The commonly used chemotherapy drugs include platinum‐based drugs such as cisplatin as well as paclitaxel. Our findings suggested that chemotherapy prolonged OS in patients with cervical MA, but did not show a significant difference in CSS. In the future, there may be a stronger focus on combination therapy regimens. With the advent of the immune era, immunotherapy may be effective for this subset of patients; however, there is a lack of evidence. Further exploration is needed to develop more precise and effective treatment options for patients with MA.

TP53 mutations are more common in non‐HPV‐related adenocarcinomas, including the gastric‐type mucinous subtype, which may contribute to their aggressive phenotype [27]. Similarly, KRAS mutations, which are common in intestinal‐type mucinous adenocarcinomas, may serve as a molecular marker to distinguish them from the usual types [28, 29]. In addition, PD‐L1 expression levels have been associated with immune evasion and may differ significantly between MA and UA, thereby affecting their response to immunotherapy [30, 31]. However, such molecular markers are not available in the SEER database and are beyond the scope of this study. Future studies integrating genomic and transcriptomic analyses will be essential to validate the utility of these biomarkers in diagnostic stratification, prognosis, and personalized treatment of endocervical adenocarcinoma.

Although conventional staging can determine prognosis, age, race, tumor size, and various treatment modalities may have an effect. Therefore, we constructed reliable nomograms to predict 1,3,5 years OS and CSS in patients with MA by screening multiple clinicopathological factors, including tumor stage, through univariate and multivariate Cox analyses. The clinical utility and predictive performance were assessed by time‐dependent ROC curves, calibration curves, and DCA curves. Multi‐dimensional results displayed that our column‐line diagrams exhibited a high degree of accuracy in comparison to conventional FIGO stage. This makes it possible to develop individualized treatment for patients with MA. In clinical practice, clinicians should actively act on those variables that can be changed by medical intervention. For example, in older patients with MA with larger tumors, surgical treatment may significantly reduce their risk score. This suggests that if such patients receive surgical treatment after ruling out surgical contraindications, their survival prognosis may be significantly improved. Therefore, the positive effect of surgical treatment on the prognosis of such patients should be fully considered in clinical decision‐making.

To our knowledge, no studies have yet investigated the construction of prognostic models for patients with cervical MA. Although this study provides a comprehensive clinical analysis, several important research gaps remain. First, the lack of molecular and genetic information in the SEER database hinders the integration of key biomarkers, such as TP53 mutations, KRAS alterations, and PD‐L1 expression, which may significantly affect prognosis and treatment response. These genetic and epigenetic features may help to further distinguish mucinous adenocarcinoma from conventional adenocarcinoma and more precisely tailor treatment. Second, due to the rarity of mucinous adenocarcinoma, prospective, multicenter studies are rare, and current treatment strategies are mainly extrapolated from other histological subtypes. Third, there is an unmet need for comprehensive prediction models that combine clinical, imaging, molecular, and treatment data to support personalized survival prediction and treatment planning. Future studies should focus on: (1) incorporating multi‐omics analyses into large cohort studies; (2) exploring the prognostic relevance of molecular markers; (3) developing decision support systems based on machine learning. These efforts will significantly enhance our ability to personalize the management of MA and UA.

In conclusion, this study found that the prognosis of MA was significantly worse than that of common adenocarcinoma through a systematic comparative study in the SEER database. To further validate this conclusion, we used PSM analyses to further confirm the poorer prognostic performance of mucinous cancer patients. On this foundation, prediction models for 1, 3, and 5 years OS and CSS were constructed for patients with MA. The models demonstrated higher predictive efficacy compared to the conventional FIGO stage system and were able to assess patients' prognosis more accurately. With this model, patients with MA can be treated and managed more precisely, which is expected to improve survival and quality of life. This study not only expands our understanding of the clinical characteristics of cervical MA, but also provides a more effective prognostic tool for clinical practice, which can help to develop more individualized treatment strategies and ultimately improve the clinical outcomes of patients.

## Author Contributions

Y.K. and L.C. proposed the main idea and drafted the initial version of the manuscript. J.L. and Y.K. conducted the data analysis and data interpretation, while L.C. and J.L. were responsible for data collection and validation. Q.X. and H.J. provided guidance on the methodology and structure of the article. Q.X. and J.L. contributed to funding acquisition. Q.X. has carefully polished and revised the overall article.

## Ethics Statement

This study was done using SEER database data, so informed consent was waived and approved by the Ethics Committee of Fujian Cancer Hospital and was conducted in accordance with the Declaration of Helsinki. All individuals signed written informed consent.

## Consent

All the authors agreed to publish this work.

## Conflicts of Interest

The authors declare no conflicts of interest.

## Supporting information


**Table S1.** Patient demographics and baseline characteristics.

**Figure S1.** Propensity score matching distribution visualized by jitter plot.

**Figure S2.** Calibration curves for external validation. (A) Calibration curves of 3‐year OS; (B) Calibration curves of 5‐year OS; (C) DCA of 3−/5‐year CSS.

**Figure S3.** DCA for external validation. Left: DCA of 3‐year OS; Right: DCA of 5‐year OS.

## Data Availability

The data supporting this study's findings are available from the corresponding author upon reasonable request.
