# Supplementary material for: Prognostic Differences and Survival Predictive Models for Mucinous Versus Usual‐Type Adenocarcinoma of the Uterine Cervix
Source: Cancer Med. 2025 May 2;14(9):e70927. doi: 10.1002/cam4.70927 (PMC12048391; doi:10.1002/cam4.70927)
Supplement: Supplementary file 1 — Table S1. Patient demographics and baseline characteristics. [file CAM4-14-e70927-s001.docx]

Table S1.Patient demographics and baseline characteristics

| **Characteristic** | **N = 65^1^** |
| --- | --- |
| **Age** |  |
| 0 | 41 (63.1%) |
| 1 | 24 (36.9%) |
| **SITE** |  |
| 0 | 28 (43.1%) |
| 1 | 37 (56.9%) |
| **T** |  |
| 0 | 4 (6.2%) |
| 1 | 14 (21.5%) |
| 2 | 21 (32.3%) |
| 3 | 16 (24.6%) |
| 4 | 10 (15.4%) |
| **N** |  |
| 0 | 29 (44.6%) |
| 1 | 21 (32.3%) |
| 2 | 15 (23.1%) |
| **M** |  |
| 0 | 59 (90.8%) |
| 1 | 6 (9.2%) |
| **FIGO** |  |
| 1 | 10 (15.4%) |
| 2 | 11 (16.9%) |
| 3 | 38 (58.5%) |
| 4 | 6 (9.2%) |
| **Tumor Size** |  |
| 0 | 18 (27.7%) |
| 1 | 18 (27.7%) |
| 2 | 29 (44.6%) |
| **Combined Stage** |  |
| 0 | 36 (55.4%) |
| 1 | 23 (35.4%) |
| 2 | 6 (9.2%) |
| **Surgery** |  |
| 0 | 36 (55.4%) |
| 1 | 29 (44.6%) |
| **Chemotherapy** |  |
| 0 | 32 (49.2%) |
| 1 | 33 (50.8%) |
| **radiotherapy** |  |
| 0 | 14 (21.5%) |
| 1 | 51 (78.5%) |
| ^1^n (%) | |
