# Supplementary figures and images for: Prognostic Differences and Survival Predictive Models for Mucinous Versus Usual‐Type Adenocarcinoma of the Uterine Cervix
Source: Cancer Med. 2025 May 2;14(9):e70927. doi: 10.1002/cam4.70927 (PMC12048391; doi:10.1002/cam4.70927)

# Distribution of Propensity Scores

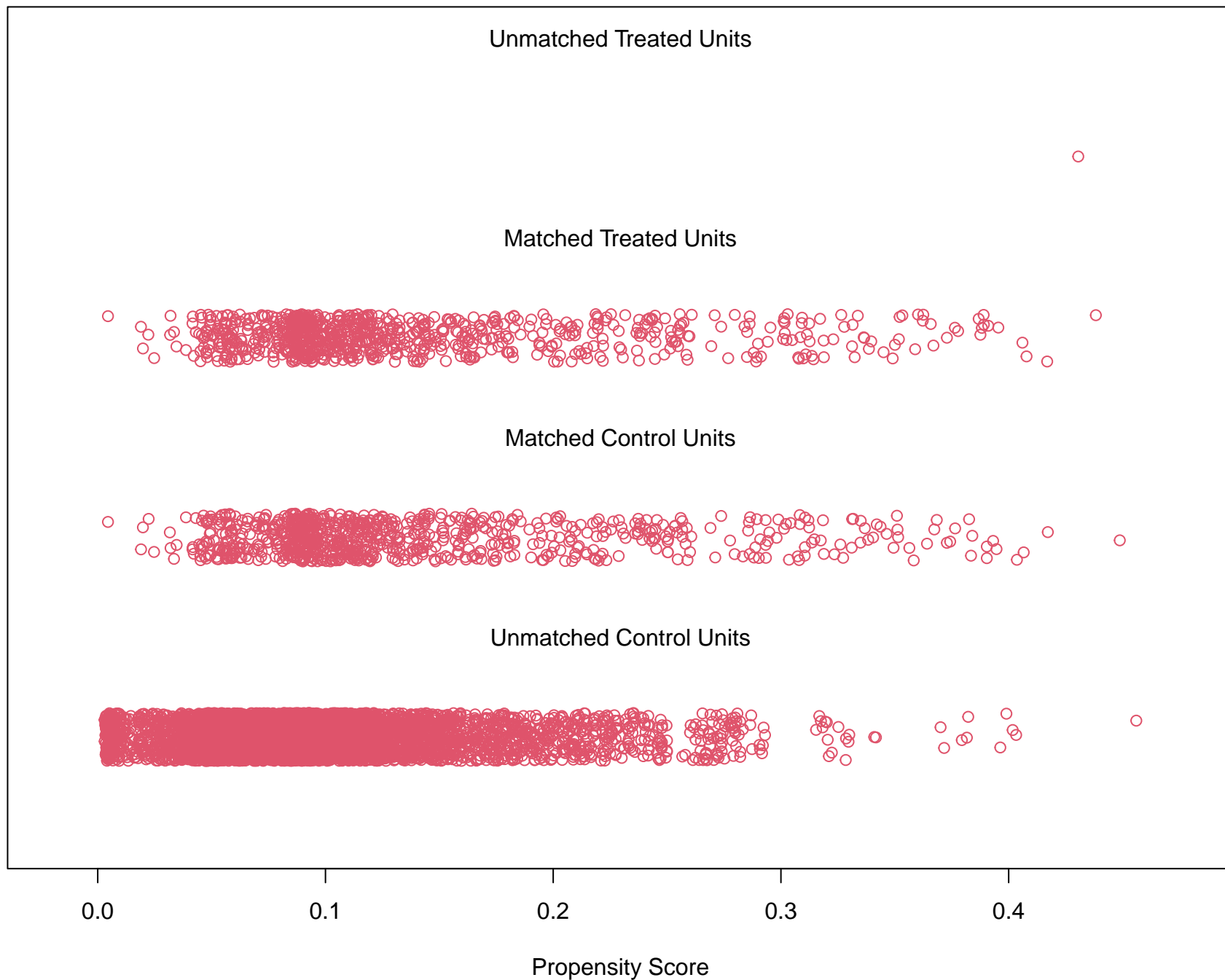

Supplement: Supplementary file 2 — Figure S1. Propensity score matching distribution visualized by jitter plot. [file CAM4-14-e70927-s004.pdf]

A

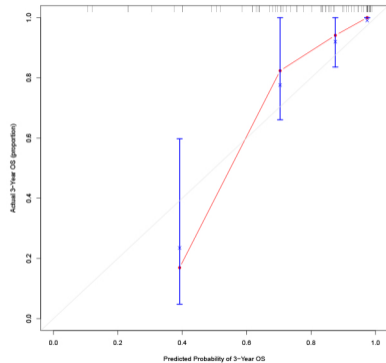

B

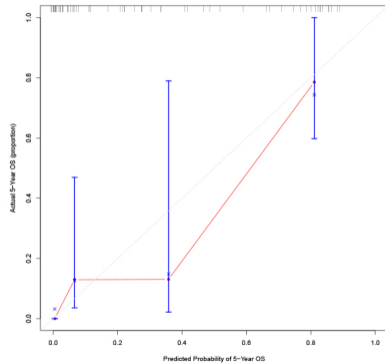

C

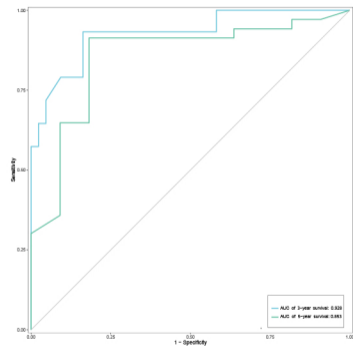

Supplement: Supplementary file 3 — Figure S2. Calibration curves for external validation. (A) Calibration curves of 3‐year OS; (B) Calibration curves of 5‐year OS; (C) DCA of 3−/5‐year CSS. [file CAM4-14-e70927-s003.pdf]

A

36

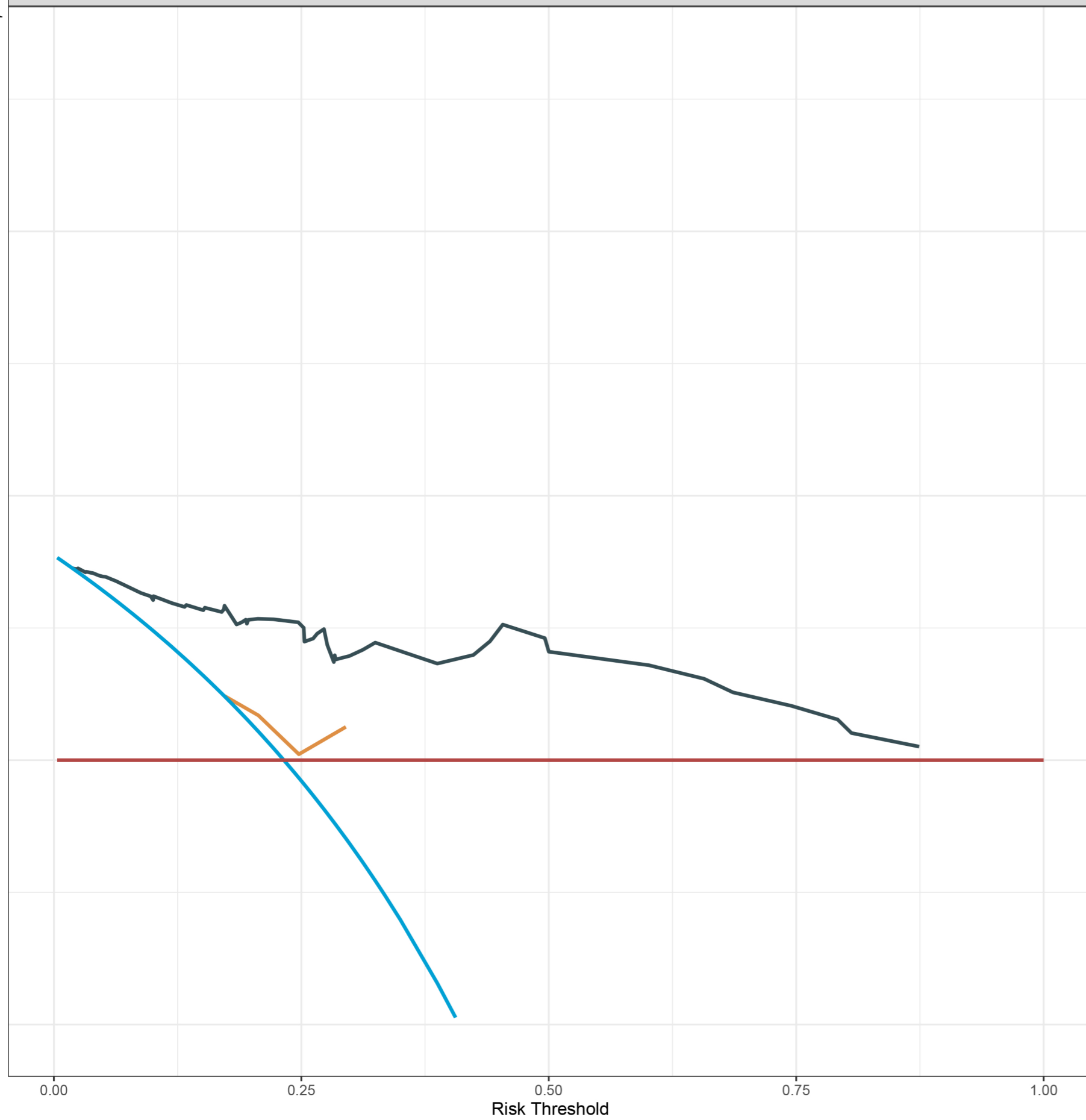

B

60

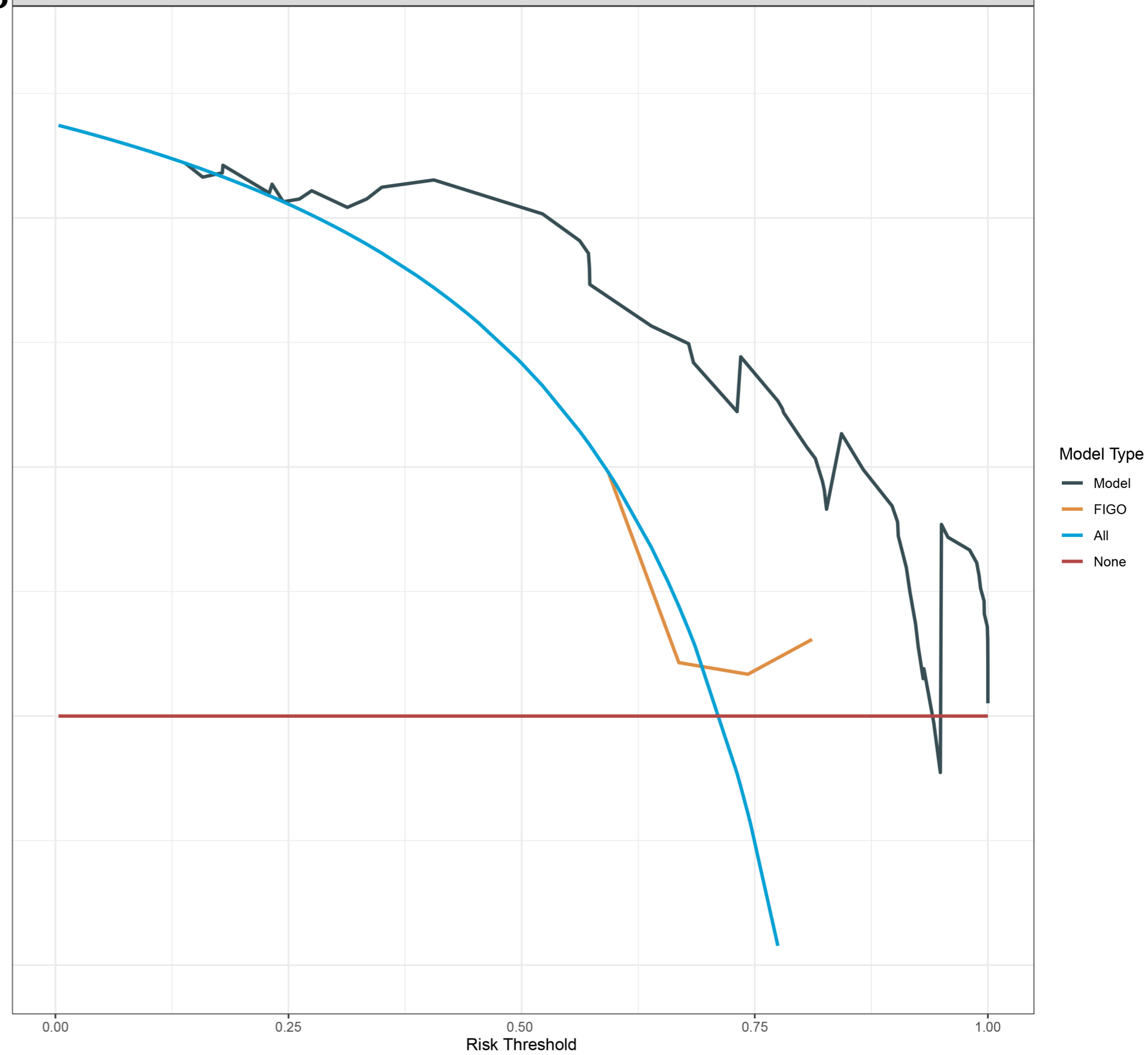

Supplement: Supplementary file 4 — Figure S3. DCA for external validation. Left: DCA of 3‐year OS; Right: DCA of 5‐year OS. [file CAM4-14-e70927-s002.pdf]
